# Osteoprotegerin Induces Apoptosis of Osteoclasts and Osteoclast Precursor Cells via the Fas/Fas Ligand Pathway

**DOI:** 10.1371/journal.pone.0142519

**Published:** 2015-11-16

**Authors:** Wei Liu, Chao Xu, Hongyan Zhao, Pengpeng Xia, Ruilong Song, Jianhong Gu, Xuezhong Liu, Jianchun Bian, Yan Yuan, Zongping Liu

**Affiliations:** 1 College of Veterinary Medicine, Yangzhou University, Yangzhou, 225009, Jiangsu, China; 2 Jiangsu Co-innovation Center for Prevention and Control of Important Animal Infectious Diseases and Zoonoses, Yangzhou, 225009, Jiangsu, China; Faculté de médecine de Nantes, FRANCE

## Abstract

Osteoprotegerin (OPG) is known to inhibit differentiation and activation of osteoclasts (OCs) by functioning as a decoy receptor blocking interactions between RANK and RANKL. However, the exact role of OPG in the survival/apoptosis of OCs remains unclear. OPG caused increased rates of apoptosis of both OCs and osteoclast precursor cells (OPCs). The expression of Fas and activated caspase-8 was increased by both 20 ng/mL and 40 ng/mL of OPG, but was markedly decreased at 80 ng/mL. Interestingly, we noted that while levels of Fas ligand (FasL) increased with increasing doses of OPG, the soluble form of FasL in the supernatant decreased. The results of a co-immunoprecipitation assay suggested that the decrease of sFasL might be caused by the binding of OPG. This would block the inhibition of the apoptosis of OCs and OPCs. Furthermore, changes in expression levels of Bax/Bcl-2, cleaved-caspase-9, cleaved-caspased-3 and the translocation of cytochrome c, illustrated that OPG induced apoptosis of OCs and OPCs via the classic Fas/FasL apoptosis pathway, and was mediated by mitochondria. Altogether, our results demonstrate that OPG induces OCs and OPCs apoptosis partly by the Fas/FasL signaling pathway.

## Introduction

Since the discovery of the first tumor necrosis factor, tumor necrosis factor alpha (TNFα), members of TNF superfamily [1] have been found, many TNF family members have shown promise in several therapeutic applications including cancer, infectious disease, transplantation and autoimmunity [2].

Osteoprotegerin (OPG), a member of the TNF family, is a secreted glycoprotein that prevents receptor activator of nuclear factor kappaB ligand (RANKL) from binding to receptor activator of nuclear factor kappa B (RANK), thereby leading to the inhibition of osteoclast differentiation and activation [3]. Since its discovery [4, 5] many studies on OPG have focused on its modulatory role in osteoclastogenesis and bone resorption [6–8]. However, whether OPG plays a role in modulating osteoclast survival/apoptosis remains less clear. Although it is known that RANKL is essential for osteoclast survival [9], and the binding of RANKL to RANK would elicit a complex signalization cascade resulting in osteoclast-specific gene transcription and survival pathway activation [10], there is no evidence that the decoy receptor role of OPG would impede the survival pathway, even lead to the apoptosis of osteoclasts (OCs).

Programmed cell death through apoptosis plays a major regulatory role in homeostasis by maintaining a balance between cell proliferation and cell death. Apoptosis helps eliminate cells that are no longer necessary for the function of tissues [11]. Therefore, apoptosis is both a normal process during development and adult tissue homeostasis and a necessary physiological cellular response to many noxious stimuli, executed by the cascade of molecular events involving a number of membrane receptors and cytoplasmic proteins [12–15]. Among the cell death receptors, the CD95/APO-1 (Fas)/Fas ligand (FasL) system provides an important apoptotic mechanism. The binding of FasL to Fas recruits Fas associated death domain (FADD), and elicits the activation of a downstream caspase (cysteine aspartic acid proteases) cascade. The mitochondrial component of the apoptotic process is mediated by truncated BH3 interacting domain death agonist (BID) translocation to the mitochondria from the cytosol and subsequent cytochrome c release [16].

It has been known for many years that the correct functioning of the immune system requires it to maintain an equilibrium. Similarly, an exquisite balance is acknowledged to be important in bone [17]. The immune system and bone are anatomically and functionally closely related, sharing common progenitor cells and various cytokine networks [18]. Furthermore, apoptosis regulates the development and function of both systems. It is well known that the Fas/FasL system is the major apoptotic mediator in the immune system, and in recent years, there have been many studies demonstrating that the Fas/FasL system has an effect on the regulation of bone turnover[19, 20] and osteoclast progenitor apoptosis [21]. In this study, we report the novel function of FasL/Fas in OCs and osteoclast precursor cells (OPCs) apoptosis induced by OPG.

## Materials and Methods

### Reagents

Penicillin, streptomycin and DAPI (4', 6-diamidino-2-phenylindole) were purchased from Sigma-Aldrich (St. Louis, MO, USA). OPG, M-CSF and RANKL were obtained from PeproTech Inc. (Rocky Hill, CT, USA). Dulbecco’s modified Eagle’s medium (DMEM), α-MEM and fetal bovine serum (FBS) were obtained from Gibco (Grand Island, NY, USA). Trypsin was obtained from Amresco (Solon, OH, USA). Antibodies against Bax, Bcl-2, cleaved-caspase-3, cleaved-caspase-9, cleaved-caspase-8, cytochrome c and β-actin were purchased from Cell Signaling Technology (Boston, MA, USA). Antibodies against Fas and Fas Ligand were purchased from Boster (Pleasanton, CA, USA). Horseradish peroxidase (HRP)-conjugated goat anti-rabbit immunoglobulin G (IgG) was purchased from Santa Cruz Biotechnology (Santa Cruz, CA, USA). Other chemicals and reagents were purchased locally at analytical grade.

### Cell Isolation and Cell Culture

The study was carried out in strict accordance with the recommendations in the Guide for the Care and Use of Laboratory Animals of the National Research Council[22]. The Animal Care and Use Committee of Yangzhou University approved all experiments and procedures conducted on the animals (approval ID: SYXK (Su) 2007–0005).

Mouse osteoclasts were generated by flushing bone marrow cells from the long bones of 8–12-week-old male Balb/cJ mice. The mice were obtained from the Laboratory Animal Center in Yangzhou University (Yangzhou, China). They has been kept at the Laboratory Animal Center in Yangzhou University, housed in a quiet, temperature-controlled room at 22°C– 23°C and were provided with water and dry food. The mice were anesthetized with xylasol/ketamine and sacrificed by cervical dislocation. After being plated overnight in complete α-MEM containing 10% FBS, 2 mM L-glutamine, 100 units/ml penicillin, 100 μg/ml streptomycin, and 10 ng/ml M-CSF, non-adherent cells were collected and layered onto a Ficoll-Hypaque gradient (GE Healthcare, NSW, Australia) and centrifuged. Bone marrow monocyte/macrophage lineage cells were collected from the interface, washed, and cultured for 5–7 days in complete α-MEM, supplemented with 25 ng/ml M-CSF + 40 ng/ml RANKL. The medium was replaced every 3 days. After 5 days cultivation, 0, 20, 40 and 80 ng/ml of OPG were added to different groups of cells, after removing RANKL. The cells were cultured with M-CSF for another 24 hours prior to each experiment.

### Study of Apoptosis

Cells were seeded in six-well flat-bottomed plates on coverslips. Primary OPCs were collected by trypsinization in 0.25% pancreatin and washed twice with phosphate-buffered saline (PBS). The apoptosis rate was determined using an Annexin V-FITC and propidium iodide (PI) double staining kit according to the manufacturer’s instructions. Briefly, cells were resuspended with binding buffer containing Annexin V-FITC and/or PI stock solution and incubated in darkness at room temperature for 15 min. We analyzed the cells using flow cytometry as described above within 1 h of treatment.

The TACS Blue Label kit (Trevigen Inc., Gaithersburg, MD) was used to detect and quantify apoptosis of primary OCs, which were retained on the coverslips after trypsinization. This TUNEL-derived method enables in situ visualization of DNA fragmentation at the single cell level by cytochemistry. Cells were fixed, washed and permeabilized with cytonin. Biotinylated nucleotides were incorporated by Terminal eoxynucleotidyl Transferase (TdT). Streptavidin-HRP conjugates were then added, followed by the substrate, TACS Blue Label. The resulting enzymatic reaction generated an insoluble blue-colored precipitate where DNA fragmentation had occurred. Thereafter, TRAP staining was conducted in accordance with the manufacturer’s instructions. The stained samples were examined using a light microscope.

### sFas Ligand ELISA

We collected the culture medium in six-well plates as described above and conducted the assay immediately. We determined the concentration of soluble Fas ligand in the medium by ELISA (detection limit: 10 pg/ml) using a commercial kit (BioVision Inc, Milpitas, CA).

### Co-Immunoprecipitations

For co-immunoprecipitation assays, cells were treated with 40 ng/mL of OPG. The cells were collected in PBS supplemented with protease inhibitors (Applygen Technologies Inc., Beijing, China) and lysed by sonication. Then, 100 μL of Protein A / Protein G SureBeads Magnetic Beads suspensions (BIO-RAD, Hercules, California) were incubated while rolling with 2 μg anti-FasL antibody (Santa Cruz, CA, USA) for 10 min at room temperature. The beads were precipitated on a magnetic rack and washed three times with PBST (PBS + 0.1% Tween 20). The lysates then were added into the magnetic beads for 1 h rolling incubation at room temperature. The beads were precipitated again and washed three times with PBST. The immunoprecipitates were separated from the beads by using Glycine elution buffer (20 mM, PH 2.0). After SDS-PAGE, the immunoprecipitates were probed with an anti-OPG antibody (Santa Cruz, CA, USA) in a Western blot analysis.

### Subcellular Fractionation

The treated cells were harvested and homogenized in ice-cold hypotonic buffer (20 mM HEPES–KOH, pH 7.5, 10 mM KCl, 1.5 mM MgCl_2_, 1 mM EDTA, 1 mM EGTA, 1 mM DTT, 0.1 mM PMSF, 250 mM sucrose) containing complete protease inhibitor cocktail. Nuclei and intact cells were removed by centrifugation at 750 g for 5 min at 4°C. The resultant supernatants were then centrifuged at 10,000 g for 30 min at 4°C, and the pellets were resuspended in SDS buffer (10 mM Tris-HCl, pH 7.4, 1 mM EDTA, 1 mM EGTA, 0.15 M NaCl, 0.5 mM PMSF, 2 mM NaVO_3_, 1% SDS) and designated as the mitochondrial fraction. The supernatant from the 10,000 g centrifugation was further centrifuged at 100,000 g for 15 min at 4°C, and the resultant supernatant was designated as the cytosolic fraction. Protein concentrations were determined by bicinchoninic acid assay with BSA as a standard prior to being stored at -80°C.

### Western Blot Analysis

Total proteins (60–80 μg) were separated on 10%–15% SDS-polyacrylamide gels and transferred to nitrocellulose membranes. The membranes were incubated with TBS containing 0.05% Tween 20 and 5% nonfat milk to block nonspecific binding. The membranes were incubated overnight at 4°C with antibodies against Fas, Fas ligand, cleaved-caspase-8, Bax, Bcl-2, cytochrome c, cleaved-caspase-3, caspase-7, cleaved-caspase-9 or β-actin (1:1,000 dilution), followed by incubation with the corresponding HRP-conjugated secondary antibodies (1:5,000 dilution). Protein detection was performed by enhanced chemiluminescence. All assays were performed in triplicate.

### Statistical Analysis

All experiments were carried out with a minimum of three independent repeats. The mean ± SD was determined by one-way ANOVA by using SPSS software. The results were considered significant at P < 0.05 and highly significant at P < 0.01.

## Result

### OPG Induces Apoptosis of Primary OCs and OPCs

**Fig 1:**
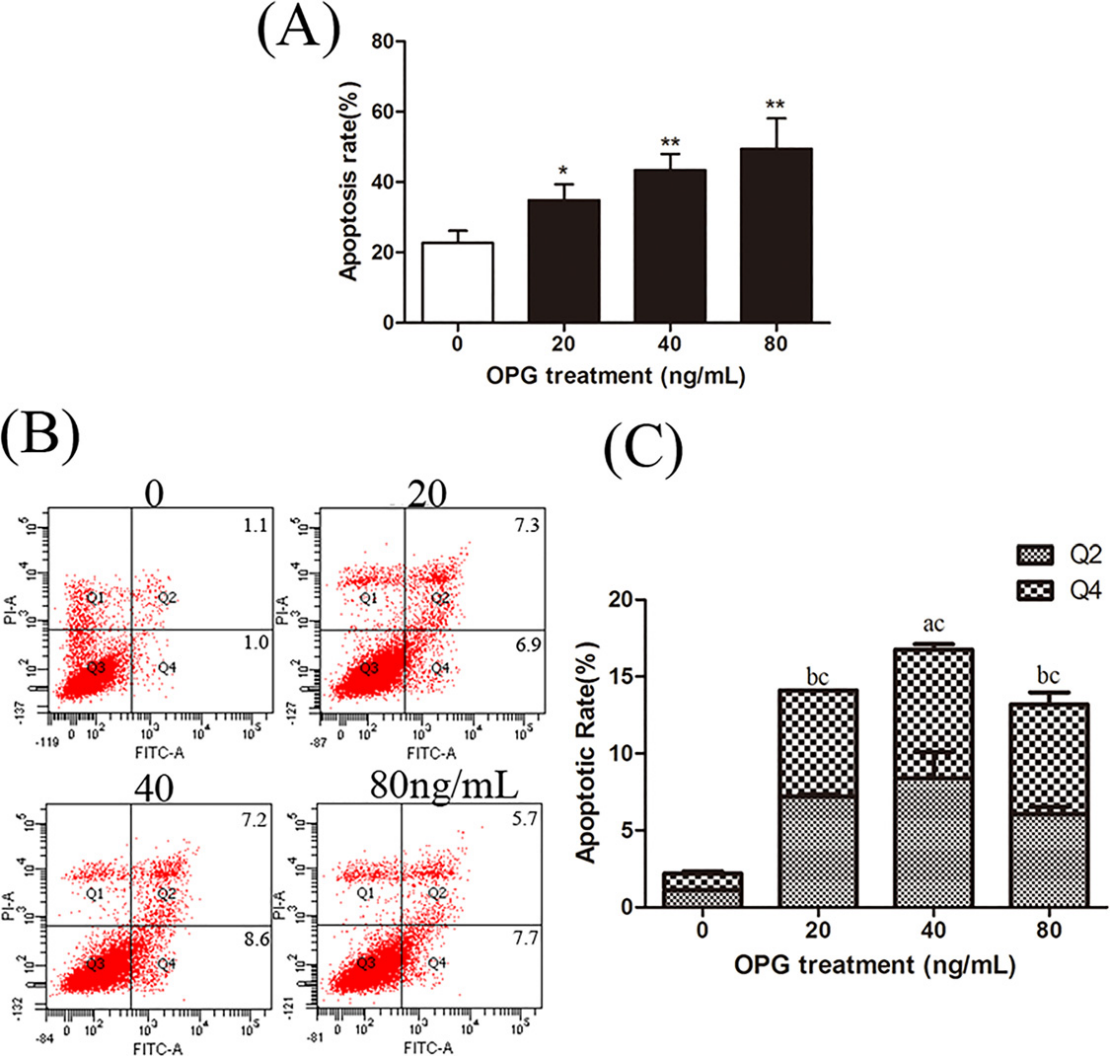
OPG induces apoptosis in primary osteoclasts (OCs) and osteoclast precursor cells (OPCs) in a dose-dependent manner. Cells deprived of RANKL had added to them various doses of OPG (20, 40, 80 ng/mL). (A) Apoptosis of OCs was evaluated after 24 h using a TUNEL-derived method. Results are expressed as the percentage of apoptotic OCs. Data are expressed as mean ± SD (n = 3) relative to control. *P < 0.05, **P < 0.01 in comparison to the control by one-way ANOVA. (B) Apoptosis of OPCs collected by trypsinization was determined by flow cytometry for Annexin-V-FITC and propidium iodide (PI) dual labeling. Cells in the Q2 and Q4 quadrants represent apoptotic cells. The mean is present in the Q2 and Q4 quadrant. (C) We calculated the population of apoptotic cell. Data are expressed as mean ± SD (n = 3) relative to control. ^a^ *P* < 0.05 and ^b^ *P* < 0.01 represent the data of Q2 in comparison to the control, ^c^ *P* < 0.01 represents the data of Q4 the in comparison to the control by one-way ANOVA.

After treatment with OPG, we evaluated the level of apoptosis of primary OCs using a TUNEL-derived method. In our control cultures, apoptosis was present in 22.7% ± 3.4% of OCs, which represented the basal level of apoptosis. The percentage of apoptotic OCs increased to 34.8 ± 4.6% in the presence of 20 ng/mL OPG (*p* = 0.021), 43.5% ± 4.6% in the presence of 40 ng/mL OPG (*p* = 0.003) and 51.3% ± 8.6% in the presence of 80 ng/mL OPG (*p* = 0.007; Fig 1A). Our results show OPG increases apoptosis significantly in our osteoclast cultures in a dose-dependent manner.

We next measured the apoptotic rate of primary OPCs by flow cytometry using Annexin V/PI double staining kit. The results revealed that both early and late stage apoptosis were augmented significantly in the presence of OPG (Fig 1B and 1C).

### Fas/FasL Participates in the OPG Induced Apoptosis of Primary OCs and OPCs

**Fig 2:**
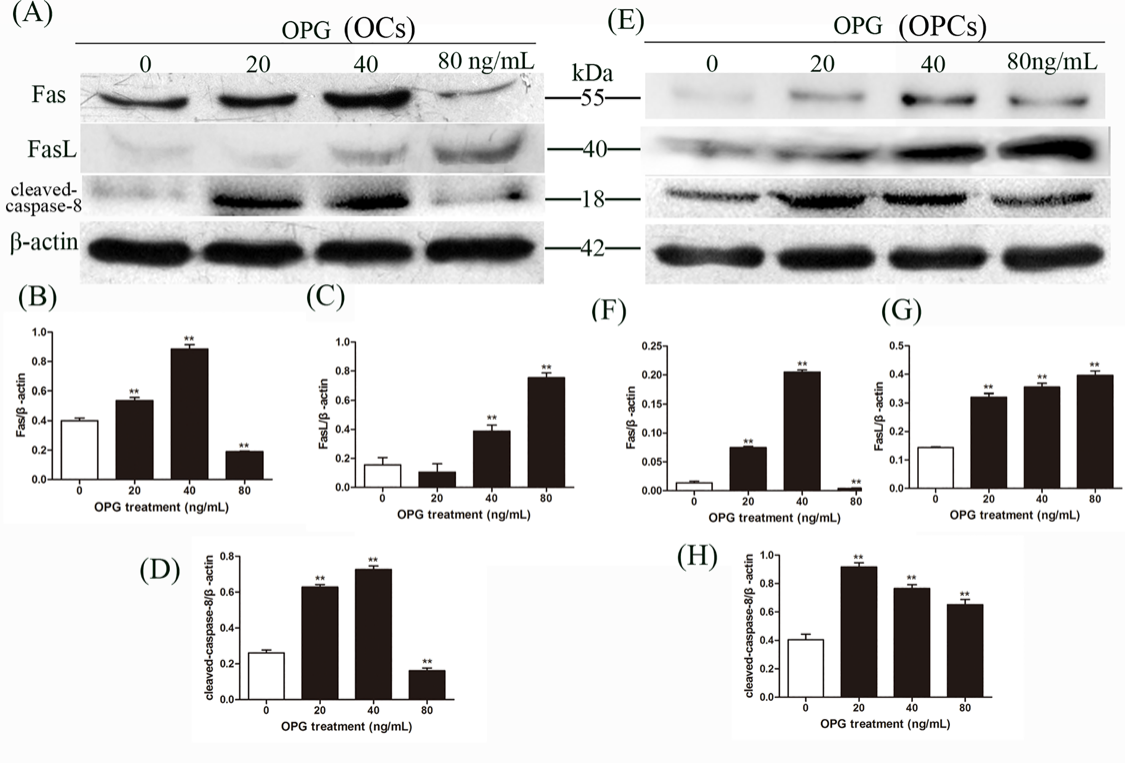
OPG activates the Fas/FasL apoptotic pathway in OCs and OPCs. At the end of culture, OCs and OPCs were harvested by corresponding methods. We evaluated Fas, FasL and cleaved-caspase-8 expression by western blotting using the relevant antibodies in OCs (A) and OPCs (E). Blots for Fas, FasL and cleaved-caspase-8 in OCs (B-D) and OPCs (F-H) were semi-quantified using Image LabTM software. Data are expressed as mean ± SD (n = 3) relative to control. **P < 0.01 in comparison to the control by one-way ANOVA.

**Fig 3:**
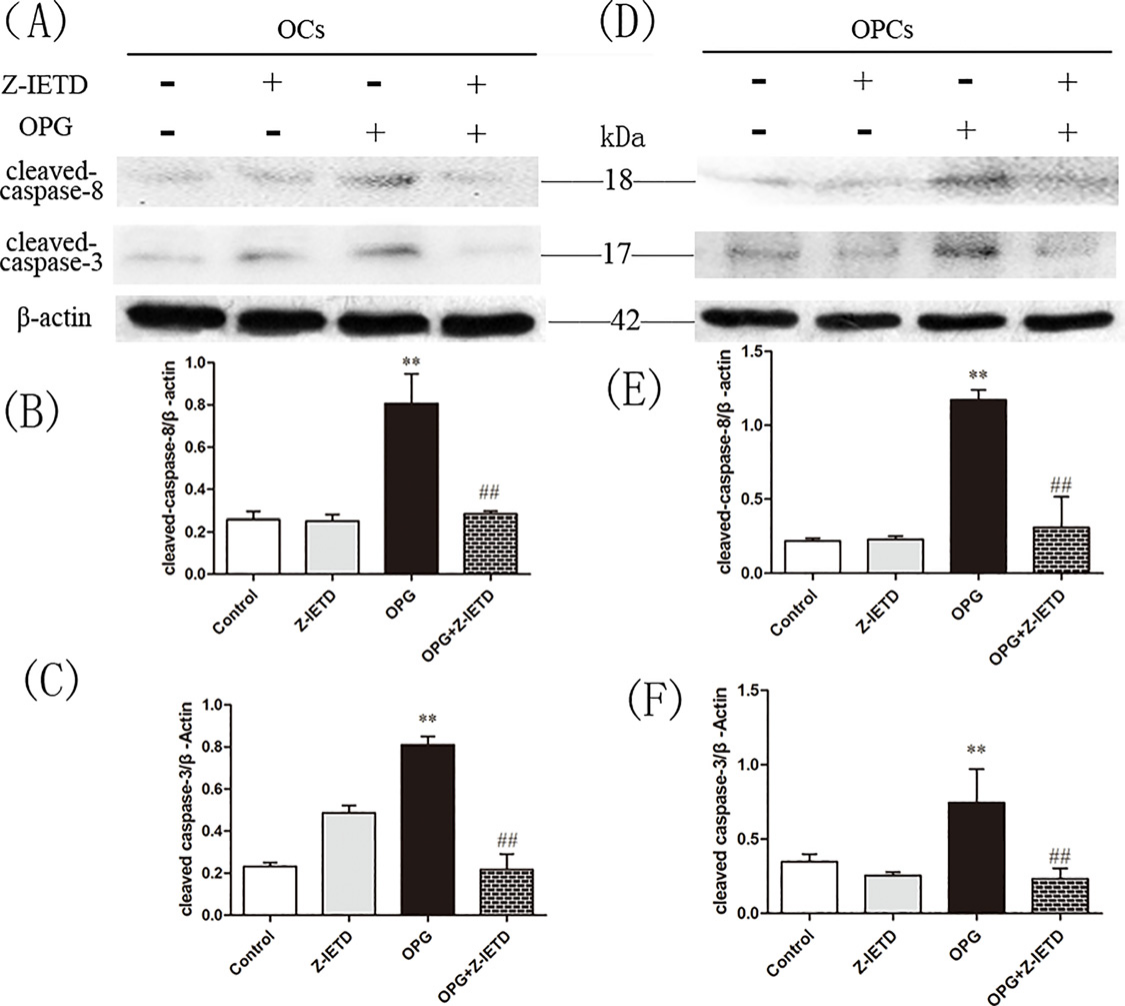
Western blotting shows that co-treatment with the caspase-8 inhibitor Z-IETD-FMK reverses OPG-induced activation of caspase-8 and caspase-3 in OCs and OPCs. Cells were pretreated with Z-IETD-FMK (40 μM) for 4 h prior to treatment with OPG (40ng/mL) for 24 h. Cleaved-caspase-8 and cleaved-caspase-3 expression in OCs (A) and OPCs (D) was detected by Western blotting using the relevant antibodies. All experiments were performed in triplicate. Blots for cleaved-caspase-8 and cleaved-caspase-3 in OCs (B and C) and OPCs (E and F) were semi-quantified using Image LabTM software. Data are expressed as mean ± SD (n = 3) relative to the control. **P < 0.01 in comparison to the control by one-way ANOVA. ^##^ *P* < 0.01 in comparison to the OPG treatment by one-way ANOVA.

To explain how OPG induces apoptosis of OCs and OPCs, we hypothesized that OPG may act by influencing the Fas/FasL system. We applied western blotting analysis using the relevant antibodies and revealed that at 20 ng/mL and 40 ng/mL OPG expression levels of Fas increased in both OCs and OPCs, while at 80 ng/mL expression levels suddenly decreased (Fig 2A, 2B, 2E and 2F). At this OPG concentration, we also noted a decrease in the levels of activated caspase-8 in OCs (Fig 2A and 2D). In OPCs, we reported an increase in the expression levels of activated caspase-8 compared with the control, which then declined in a dose-dependent manner (Fig 2E and 2H). Meanwhile, in the presence of OPG in both OCs and OPCs, the expression levels of FasL increased in a dose-dependent manner (Fig 2A, 2C, 2E and 2G). Furthermore, we established that pretreatment with Z-IETD-FMK (40 μM) for 4 h prior to treatment with OPG (40 ng/mL) for 24 h was sufficient to inhibit apoptosis in OCs and OPCs, as indicated by Western blotting (Fig 3). These findings suggest that in both OCs and OPCs, Fas/FasL and its downstream proteins are involved in apoptosis induced by OPG.

### OPG Induces Apoptosis of OCs by Limiting the Soluble Form of FasL in the Supernatant

**Fig 4:**
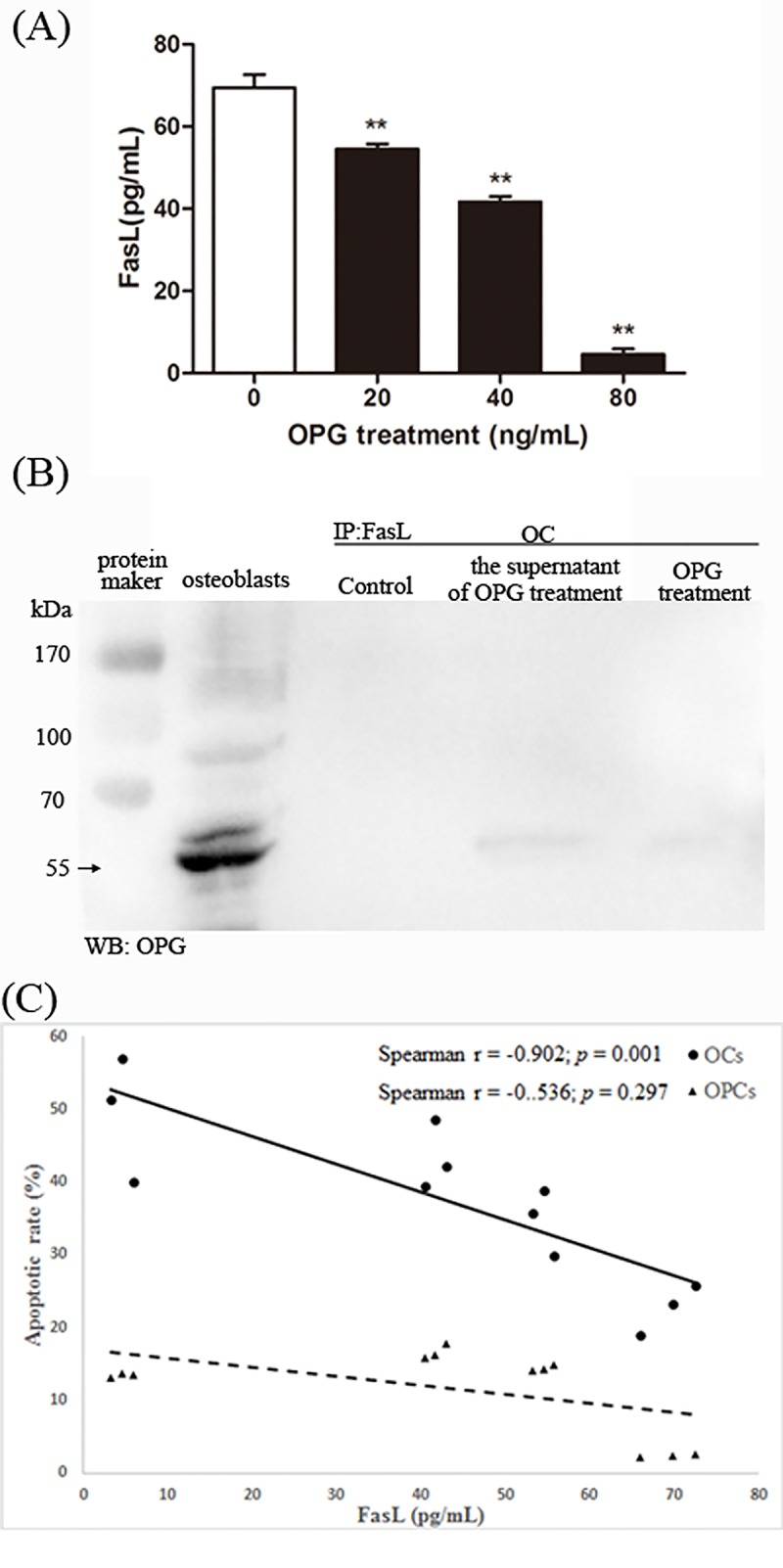
OPG induces apoptosis of OCs by binding the soluble form of FasL in the supernatant. At the end of the culture, OPG was added at different doses, after removal of RANKL. (A) sFasL concentrations were measured by ELISA in the culture media 24 h later (n = 3) (**P < 0.01) (B) OCs and OPCs were treated with 0 and 40 ng/mL OPG for 24 h. Supernatants and OCs/OPCs were subjected to immunoprecipitation with polyclonal anti-FasL (IP FasL) followed by immunoblotting with OPG antibodies. The lysate of osteoblasts was used as the positive control for OPG expression. (C) In the three experiments, the apoptotic rate of OCs and OPCs was evaluated by corresponding methods. A correlation analysis between sFasL concentrations in the culture media and the percentage of apoptotic OCs and OPCs detected in the same cultures, was performed with Spearman test and linear regression.

We used FasL ELISA to quantitatively determine the concentration of soluble FasL in our osteoclast cultures. We noted the concentration of FasL in the osteoclasts control group as 69.44 ± 3.22 pg/mL, which then significantly decreased when we added increasing doses of OPG. We recorded the level of FasL as 54.51 ± 1.28 pg/mL in the presence of 20 ng/mL OPG, 41.71 ± 1.55 pg/mL in the presence 40 ng/mL OPG and 4.59 ± 1.29 pg/mL in the presence of 80 ng/mL OPG (P < 0.01; Fig 4A).

Co-immunoprecipitation assays were conducted to investigate the interaction between OPG and sFasL, cell-surface FasL. We found that OPG would bind to both sFasL in the surpernate and the membrane-bound FasL (Fig 4B). However, the binding of sFasL was more effective than that of cell-surface FasL.

To further relate the apoptosis of OCs and OPCs to the shedding of FasL, we analyzed the correlation between sFasL production in the culture media and the rate of apoptosis of OCs and OPCs. We reveal increasing OPG concentrations resulted in increasing apoptosis of OCs and OPCs relative to control cells; the concentration of sFasL decreased in the same dishes. Statistical analyses revealed a high correlation coefficient between the rate of apoptosis of OCs and sFasL production (-0.902), which was highly significant (*P* = 0.001). In addition, the correlation coefficient between the rate of apoptosis of OPCs and sFasL production (-0.536) was lower with no significance (*P* = 0.297; Fig 4C). These data suggested that OPG may induce apoptosis of OCs by binding the soluble form of FasL in the supernatant.

### OPG Induces Apoptosis via the Classic Fas/FasL Apoptosis Pathway in OCs and OPCs

**Fig 5:**
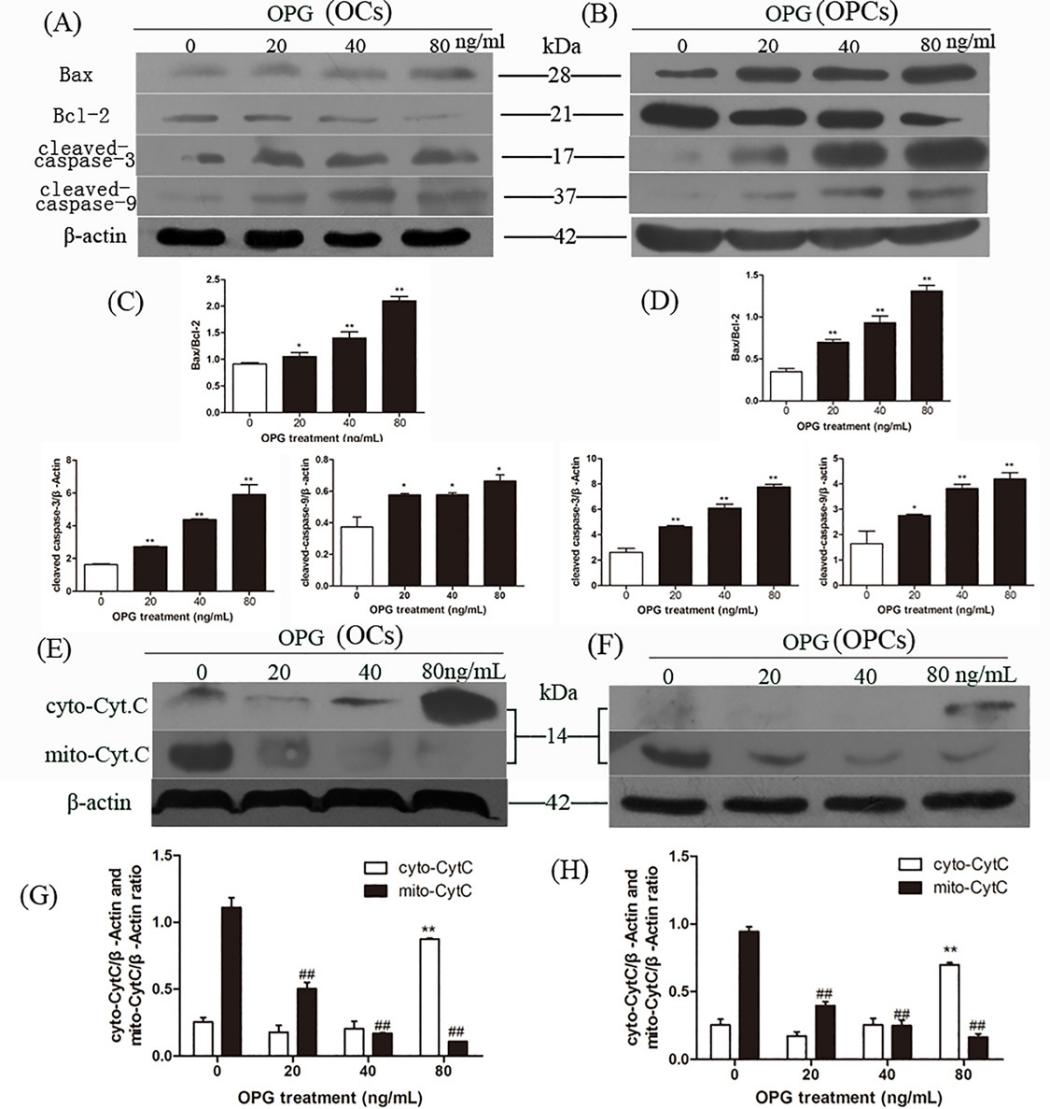
OPG induces apoptosis via the classic Fas/FasL apoptosis pathway in OCs and OPCs. At the end of the cultures, OCs and OPCs were harvested by corresponding methods. Bax, Bcl-2, cleaved-caspase-3 and cleaved-caspase-9 expression was evaluated by western blotting using relevant antibodies in OCs (A) and OPCs (B). Blots for Bax, Bcl-2, cleaved-caspase-3 and cleaved-caspase-9 in OCs C) and OPCs (D) were semi-quantified using Image LabTM software. The levels of cytochrome c compared to β-actin were determined by Western blotting prior to immunostaining and fluorescence microscopy of the mitochondria and cytosol of OCs (E) and OPCs (F). Blots for cytochrome c in OCs (G) and OPCs (H) were semi-quantified using Image LabTM software. Data are expressed as mean ± SD (n = 3) relative to control. *P < 0.05, **P < 0.01 in comparison to the control by one-way ANOVA.

To explore the mechanism by which OPG induces apoptosis of OCs and OPCs and illuminates the Fas/FasL pathway, we applied western blotting to detect any apoptosis-related proteins and Fas/FasL signaling pathway-related protein expression. In both OCs and OPCs, we found a significant increase in the Bax/Bcl-2 ratio with increasing OPG concentrations. We also noted an increase in the level of activated caspase-3 and cleaved-caspase-9 with increasing doses of OPG (Fig 5A–5D). In addition, for both OCs and OPCs, OPG caused the release of cytochrome c from the mitochondria into the cytosol (Fig 5E–5H). Therefore, our findings indicate that OPG induces apoptosis of OCs and OPCs by triggering the downstream mitochondria caspase cascade of the Fas/FasL pathway.

## Discussion

Bone remodeling is a constant process that relies in the resorption role of OCs and the formation role of osteoblasts. Dysregulation of osteoclast differentiation, activity or function leads to various bone diseases including osteoporosis, osteopetrosis or rheumatoid arthritis [23]. Many hormones, cytokines and ions produced by osteoblastic cells are thought to regulate osteoclast differentiation. In our study, we focused on the regulation of OC apoptosis, which has recently been recognized as a critical regulatory factor in bone remodeling [24]. While it is assumed that the inhibition of bone resorption of OCs induced by pharmaceuticals, such as bisphosphonates and vitamin K2, would at least partly cause osteoclast apoptosis [25–27], the key regulatory mechanisms of osteoclast apoptosis have not been fully identified and characterized.

In our in vitro murine osteoclast differentiation models, developed using marrow monocyte/macrophage lineage cells after long-term culture, we found that OPG induces apoptosis in OCs and OPCs, which is consistent with the effect that OPG leads to bone resorption (Fig 1). Interestingly, in recent years, apoptosis of OCs has been considered an important therapeutic target in the treatment of bone disease [28–30]. Meanwhile, OPG is considered a major agent in influencing bone resorption of OCs. Yet it remains unclear exactly how OPG regulates apoptosis of OCs. In contrast to our study, Estelle et al. reported OPG inhibits apoptosis of OCs by binding TNF-related apoptosis-inducing ligand (TRAIL) [31]. Nevertheless, there is also evidence showing that in vivo OPG does not block TRAIL, because TRAIL does not seem to have a direct effect on osteoclast formation or osteoclast activity in vitro or in vivo [32–34]. In our study, we found OPG treatment without RANKL led to the apoptosis of OCs and OPCs. This mechanism provides a potential death signal for previously formed OCs.

Fas, a far better characterized death receptor family, has been reported to play an important role in regulating the death of the immune system through activation-induced cell death [35]. Previous findings demonstrated that Fas and FasL are expressed during osteoclast differentiation [21, 36, 37], and the Fas/FasL system induces apoptosis in mature osteoclasts [36]. Studies on the role of Fas/FasL in bone homeostasis in vivo further clarified that functional ablation of FasL in gld mice increases bone mass and decreases osteoclasts [38], while functional ablation of Fas in gld and lpr mice decreases bone mineral density and increases osteoclast numbers [39]. Here, we reveal a novel apoptotic action of the Fas/FasL system, which is induced by OPG (Fig 2). A recent study showed that osteoblasts induced osteoclast apoptosis by the Fas/FasL pathway [40]. However, to date no study has investigated the action of OPG on the Fas/FasL system of osteoclasts. We found that only low concentrations of OPG activated the Fas/FasL pathway of osteoclast apoptosis. This result confirms the former study that the endogenous FasL does not have a major role in apoptosis [41]. Furthermore, sFasL is less efficient than the membrane form [42], the level of sFasL in the culture medium decreased sharply (Fig 4A), while the expression of FasL on membrane increased. Although the exact effect of sFasL was controversial, there were many studies showing that sFasL is a less potent inducer of apoptosis than its membrane-bound form [42–44]. The shedding of FasL from the membrane is a mechanism for downregulating at least part of its killing activity, we assumed that the downregulation of Fas/FasL-induced apoptosis might exert by the competition between sFasL and membrane-bound FasL for binding of Fas. In doing so, OPG might induced OCs apoptosis through binding sFasL (Fig 4B), or inhibiting the shedding of FasL. These results combined with the correlation analyses (Fig 4C) suggest that OPG might induce OCs apoptosis by regulating the form of FasL.

Wu et al. [39] demonstrated that the binding of Fas by anti-Fas antibody promotes apoptosis of OCs and is mediated by the classical Fas signaling including the activation of caspases 3 and 9 and the release of cytochrome c from mitochondria. In this study, apoptosis of OCs and OPCs induced by OPG was mediated by the classic Fas/FasL pathway, which in turn was activated by cleaved caspase-3 and caspase-9. Furthermore, the release of cytochrome c from mitochondria was involved in the apoptosis of OCs and OPCs (Fig 5).

**Fig 6:**
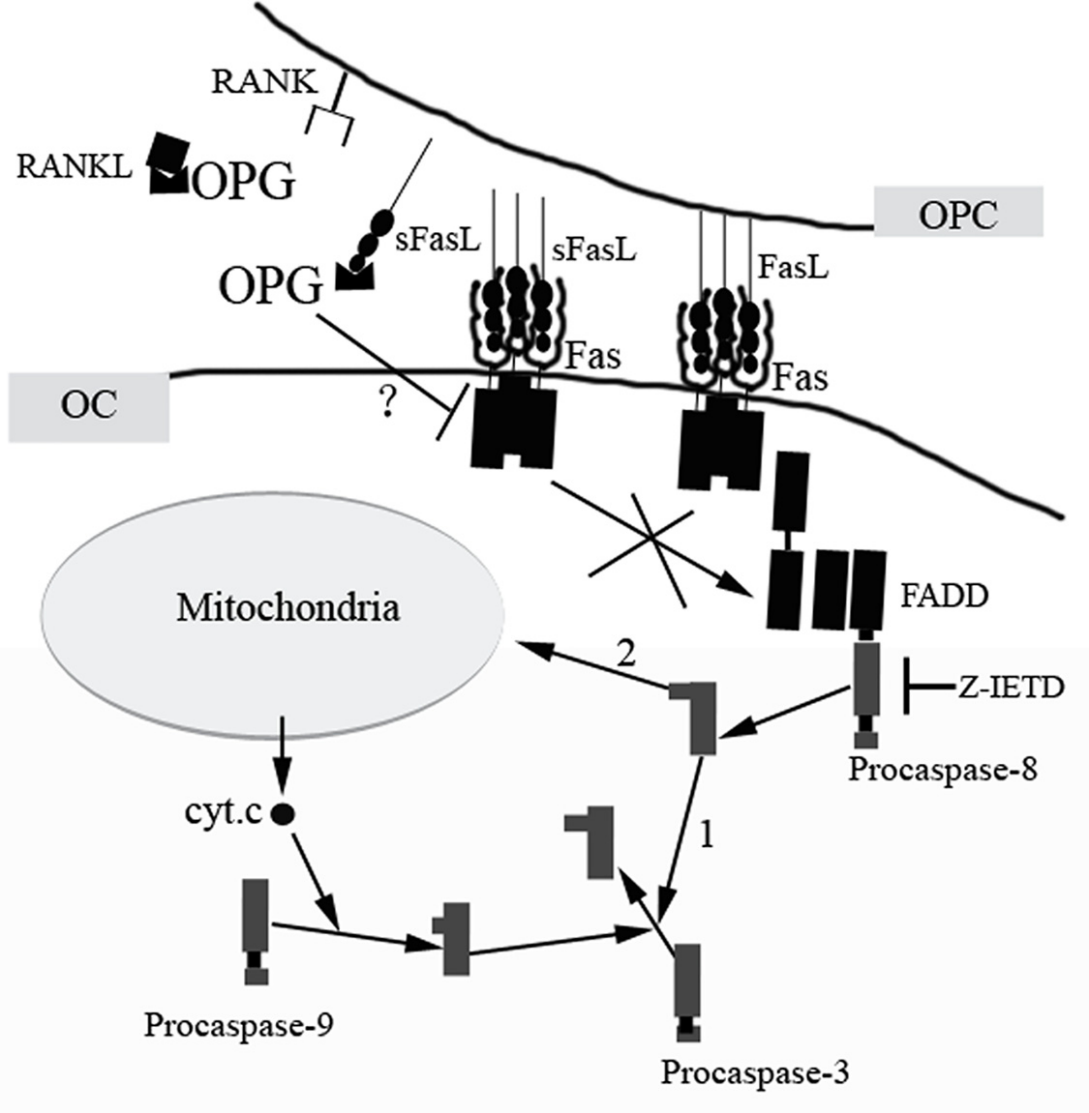
Schematic representation of the proposed OPG-induced pathway in OCs and OPCs. OPG induces Fas-mediated apoptotic signal transduction pathways by binding to sFasL, or by downregulating the shedding of membrane-bound FasL. FasL induces the clustering of intracellular death domains that recruit FADD. FADD, in turn, recruits the upstream procaspase-8 to the receptor complexes via a DED–DED interaction. As procaspase-8 has substantial enzymatic activity, recruited procaspases can be cleaved. Activated caspase-8 can directly cleave procaspase-3 (pathway 1) or can activate the mitochondria-dependent pathway of apoptosis (pathway 2).

In conclusion, our investigations reveal that OPG promotes apoptosis of OCs and OPCs by activating the Fas/FasL pathway (Fig 6). In addition, OPG-induced Fas/FasL activation regulates osteoclast apoptosis using any cell-type-specific signaling pathways. Such pathways could provide novel designs of osteoclast-specific therapeutic interventions in bone disorders. Therefore, future research is required on the precise Fas-mediated intracellular signaling pathways in osteoclast apoptosis induced by OPG.

## Supporting Information

S1 Fig RANKL residue detection After treated, immunostaining was performed with RANKL (red) antibody and cell nuclei were simultaneously stained with DAPI (blue). Cells were viewed by fluorescence microscopy. There were no RANKL residue in OCs and OPCs.
(TIF)
