# Supplementary figures and images for: Osteoprotegerin Induces Apoptosis of Osteoclasts and Osteoclast Precursor Cells via the Fas/Fas Ligand Pathway
Source: PLoS One. 2015 Nov 16;10(11):e0142519. doi: 10.1371/journal.pone.0142519 (PMC4646684; doi:10.1371/journal.pone.0142519)

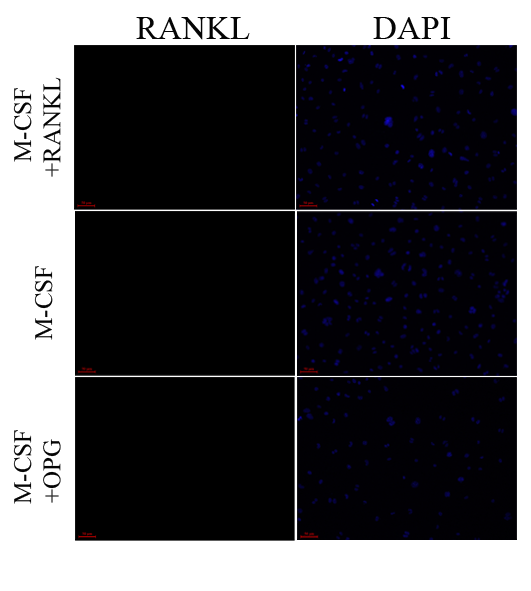

Supplement: S1 Fig — Cells were viewed by fluorescence microscopy. There were no RANKL residue in OCs and OPCs. (TIF) [file pone.0142519.s001.tif]
